# Promotion of *Para*-Chlorophenol Reduction and Extracellular Electron Transfer in an Anaerobic System at the Presence of Iron-Oxides

**DOI:** 10.3389/fmicb.2018.02052

**Published:** 2018-08-30

**Authors:** Xinbai Jiang, Yuzhe Chen, Chen Hou, Xiaodong Liu, Changjin Ou, Weiqing Han, Xiuyun Sun, Jiansheng Li, Lianjun Wang, Jinyou Shen

**Affiliations:** ^1^Jiangsu Key Laboratory of Chemical Pollution Control and Resources Reuse, School of Environmental and Biological Engineering, Nanjing University of Science and Technology, Nanjing, China; ^2^School of Chemistry and Chemical Engineering, Nantong University, Nantong, China

**Keywords:** wastewater treatment, anaerobic reduction, iron oxides, chlorophenols, extracellular electron transfer

## Abstract

Anaerobic dechlorination of chlorophenols often subjects to their toxicity and recalcitrance, presenting low loading rate and poor degradation efficiency. In this study, in order to accelerate *p*-chlorophenol (*p*-CP) reduction and extracellular electron transfer in an anaerobic system, three iron-oxide nanoparticles, namely hematite, magnetite and ferrihydrite, were coupled into an anaerobic system, with the performance and underlying role of iron-oxide nanoparticles elucidated. The reductive dechlorination of *p*-CP was notably improved in the anaerobic systems coupled by hematite and magnetite, although ferrihydrite did not plays a positive role. Enhanced dechlorination of *p*-CP in hematite or magnetite coupled anaerobic system was linked to the obvious accumulation of acetate, lower oxidation–reduction potential and pH, which were beneficial for reductive dechlorination. Electron transfer could be enhanced by Fe^2+^/Fe^3+^ redox couple on the iron oxides surface formed through dissimilatory iron-reduction. This study demonstrated that the coupling of iron-oxide nanoparticles such as hematite and magnetite could be a promising alternative to the conventional anaerobic reduction process for the removal of CPs from wastewater.

## Introduction

As one class of the most important raw materials and intermediates, chlorophenols (CPs) are widely used in many industries such as wood preservers, dyes, drugs, and herbicides (Van Aken et al., 2015). Improper management of industrial wastes, accidental spills, and liberal use of pesticides in crops have resulted in the prevalence of CPs in natural waters and soil (Garbou et al., 2017). The persistence and toxicity of CPs have incited a public awareness, causing them to be on the list of the 11 priority phenol derivatives by the United States Environmental Protection Agency (Solanki and Murthy, 2011). Hence, there is an urgent need to remediate sites polluted by CPs, in order to prevent their further risk to ecosystems.

Due to the pronounced electron-withdrawing character of chlorine substituent group, CPs harbor a highly electron deficient π-electron system on benzene ring. As a result, electrophilic attack, which is usually the first step in oxidative degradation, becomes more difficult (Czaplicka, 2006). Thus, CPs are subject to initial reductive transformation, i.e., dechlorination, although aromatic C-Cl bond is relatively stable (Duan et al., 2016). Through dechlorination, toxicity of CPs can be signally reduced because reduction products such as phenol are more readily biodegradable (Annachhatre and Gheewala, 1996; Chen et al., 2017a,b). Recently, various dechlorination technologies, such as heterogeneous catalysis (Su et al., 2011; Nascimento et al., 2016), electrochemical reduction (Arellano-González et al., 2016) and mechanochemical treatment (Deng et al., 2017), has been widely investigated. These physico-chemical methods have proven to be costly and energy intensive, and thus difficult for application at industrial scale (Liang et al., 2018). Biological reductive dechlorination, which is thought to be environmentally friendly and cost-effective, has turned out to be a favorable alternative (Limam et al., 2016). However, due to the recalcitrant and toxicological nature of pollutants such as CPs, biological degradation is often limited by low degradation rate and poor stability (Bae et al., 2017; Jiang et al., 2018; Wu et al., 2018). Lea-Smith et al. (2016) indicated that enhancement of extracellular electron transfer (EET) would be beneficial for biological reductive dechlorination, which is an electrophilic reductive reaction occurred in aqueous phase. Nevertheless, how to improve EET efficiency for biological reductive dechlorination presents an imperative but also challenging task.

Various iron-oxides, such as hematite, magnetite, and ferrihydrite, have been widely applied in wastewater remediation for the removal of heavy metal (Lin et al., 2017), dissolved sulfide (Zhang et al., 2016), and radionuclides (El Afifi et al., 2016). Recently, attention has been increasingly paid to the combined utilization of various iron-oxides into the anaerobic wastewater treatment system. Kato et al. (2012) found that the lag time could be significantly shortened and methane production rate could be increased with the addition of hematite and magnetite into anaerobic digesters. Iron-oxide nanoparticles such as hematite and magnetite stimulated the growth of *Geobacter* by syntrophic association with methanogens via enhanced electric currents. Enhanced methanogenesis in a continuous anaerobic bioreactor with magnetite supplementation was also observed by Baek et al. (2016). Hematite nanoparticles were coupled into an anaerobic system by Gadhe et al. (2015), where both glucose utilization rate and H_2_ production rate was significantly enhanced. Yao et al. (2017) and Yin et al. (2017) indicated that EET in the anaerobic treatment system could be enhanced at the presence of iron oxide. Liu et al. (2015) found that direct interspecies electron transfer could be simulated by compensation of magnetite materials in the absence of electrically conductive pili and *c*-type cytochrome. Zhuang et al. (2015) found that direct interspecies electron transfer could be enhanced at the presence of iron oxide, which could be attributed to more electron shuttles provided by iron ions released from hematite and magnetite. Introduction of conductive carbon materials could be metabolically more favorable since these conductive additives may alleviate the energy investment by microbes for the synthesis of these conductive pili and enhance EET in the meantime (Zhao et al., 2015). Therefore, it is likely that some microbes involved in biological reductive dechlorination can accomplish interspecies electron transfer mediated by electric currents through iron-oxides. However, coupling of various iron-oxides into anaerobic system for reductive dechlorination has not been reported previously, although the key role of iron oxides in anaerobic system in terms of enhancing EET has been revealed (Abdelsalam et al., 2017; Ghazzal et al., 2017). The mechanisms involved in enhanced biological reductive dechlorination through iron oxides has not yet been fully understood.

Therefore, this study aimed at investigating the feasibility of coupling iron-oxide nanoparticles into anaerobic systems for efficient reductive dechlorination. Hematite, magnetite and ferrihydrite were prepared and selected as the model iron-oxides. The performance of these iron-oxides in anaerobic system in terms of *p*-CP reduction was investigated. In addition, the underlying mechanism in terms of EET involved in enhanced *p*-CP reduction at the presence of iron-oxides was revealed.

## Materials and Methods

### Synthesis of Iron-Oxides

In this study, hydrothermal method was engaged with slight modification to synthesize polyhedral hematite nanoparticles (Zhu et al., 2012). FeCl_3_⋅6H_2_O (1.08 g), sodium acetate (2.05 g), and polyvinyl pyrrolidone (0.75 g) were dissolved into 30 mL deionized water under stirring at 25°C to form a homogeneous solution. Then the obtained solution was transferred into a stainless-steel autoclave, sealed and heated at 200°C for 5 h to get red muddy precipitate. Octahedral-shaped magnetite nanoparticles were synthesized via a modified one-pot hydrothermal route according to Lei et al. (2017). Typically, FeCl_3_⋅6H_2_O (0.3 g), sodium acetate (0.4 g), and 10 mL polyethylene glycol-200 were dissolved into 50 mL deionized water under stirring at 25°C to form a homogeneous solution. Then the obtained solution was transferred into a stainless-steel autoclave, sealed and heated at 200°C for 5 h to get black precipitate. Needle-shaped ferrihydrite nanoparticles were prepared following a modified procedure reported by Pariona et al. (2016). Typically, 6 mL sodium hydroxide (6.0 M) and 100 mL FeCl_3_ (0.3 M) were mixed together under constant stirring at 25°C to form a homogeneous solution. Then sodium hydroxide (1.0 M) were added into the solution drop by drop until precipitate was observed. Finally, the obtained hematite, magnetite and ferrihydrite precipitate was washed with deionized water and ethanol, and dried in a vacuum at 75°C for 5 h.

### Synthetic Wastewater and Sludge Acclimatization

The seed sludge was collected from an anaerobic digester in a municipal wastewater treatment plant located in Nanjing, China. Firstly, the seed sludge was filtered through a 0.9 mm stainless steel mesh for the removal of large fragments. Then the obtained sludge was acclimated in an up-flow anaerobic sludge blanket (UASB), which was operated at 35 ± 2°C at hydraulic retention time (HRT) of 24 h. The synthetic wastewater used as UASB influent was prepared as follows: NaHCO_3_ (1500 mg/L), NH_4_Cl (270 mg/L), K_2_HPO_4_⋅3H_2_O (210 mg/L), MgCl_2_⋅6H_2_O (100 mg/L), CaCl_2_ (50 mg/L), and trace element solution SL-4 (10 mL/L) prepared according to Shen et al. (2009). *p*-CP and glucose were added into the synthetic wastewater at desired concentrations. After acclimatization for about 2 months, the acclimatized sludge could be used as the inocula for batch experiment.

### Experimental Procedure for *p*-CP Reduction

Reductive dechlorination of *p*-CP was performed in batch experiment, which were carried out in a series of 120 mL glass serum vials with working volume of 100 mL. Firstly, the acclimatized sludge was washed with 0.1 M NaCl solution several times to remove residual *p*-CP and other soluble substances. Each serum vial was fed with 20 mL inoculum, and 80 mL synthetic wastewater containing 0.194 mmol/L *p*-CP and 3.47 mmol/L glucose, resulting initial *p*-CP concentration of 0.156 mmol/L, initial glucose concentration of 2.78 mmol/L and initial mixed liquor suspended solid (MLSS) concentration of 2.7 g/L. According to Zhuang et al. (2015), the dosage of iron-oxide nanoparticles was set at 100 mg/L. Serum vials with the addition of autoclaved seed sludge but without iron-oxides, serum vials with the addition of iron-oxides but without the seed sludge, and serum vials with the addition of seed sludge but without the dose of iron-oxides were used as the biomass adsorption control group, iron-oxides adsorption control group and biotic control group, respectively.

To ensure anaerobic conditions, the residual dissolved oxygen was removed by sparging the synthetic wastewater with nitrogen gas for at least 30 min. After sealing the vials with polytetrafluoroethylene plugs and aluminum covers, all serum vials were incubated on a rotary shaker at 35°C and 200 rpm. At each scheduled sampling time, one serum vial was sampled and sacrificed. The water samples taken from the sacrificed serum vials were passed through a 0.22 μm filter membrane for further analysis. All the batch experiments were performed in triplicate, with average values and standard deviation calculated.

### Analytical Methods

The SEM analysis of iron-oxides nanoparticles were characterized by a scanning electron microscope (SEM) (Quanta 250FEG, FEI, United States). For TEM analysis, samples were prepared by dispersing the nanoparticles in ethanol and dropped onto 300 mesh holey lacey carbon grids on copper supports, and then observed on a transmission electron microscope (TECNAI G2 20 LaB6, FEI, United States) operated at 350 kV. To evaluate the structural properties of the synthesized nanoparticles, X-ray diffraction (XRD) analysis was recorded on an X-ray diffractometer (D8 ADVANCE, Bruker, Germany).

*p*-CP and its dechlorination product was identified and quantified by high performance liquid chromatography (HPLC) (Waters 2695, Waters, United States), according to Wang et al. (2018). The mobile phase was a mixture of water and methanol (4/6, v/v) pumped at a flow rate of 1.0 mL/min. The injection volume for each sample was 10 μL. A water symmetry RP18 column (5 μm, 4.6 mm × 250 mm) was used for reversed-phase separation, and detection was spectrophotometric at 275 nm for *p*-CP and 280 nm for phenol. The acetic ion contents in liquid samples were analyzed using an ion chromatograph (ICS-2100, DIONEX, United States), which was equipped with an inhibitory conductance detector and an IonPac^®^ As11-HC column (4 mm × 250 mm). Hydrogen and methane contents were analyzed according to our previous work (Ou et al., 2015). Oxidation–reduction potential (ORP) and pH values were determined using a pH meter (FE20K, Mettler-Toledo Instruments, CH) with a redox electrode. Ferrous ion concentration was determined by the *o*-phenanthroline spectrophotometry. An atomic absorption spectrometer (PinAAcle900T, PerkinElmer, Waltham, MA, United States) was employed to measure the concentration of total iron.

## Results and Discussion

### *p*-CP Dechlorination in Iron-Oxide Nanoparticles Coupled Anaerobic Systems

**FIGURE 1:**
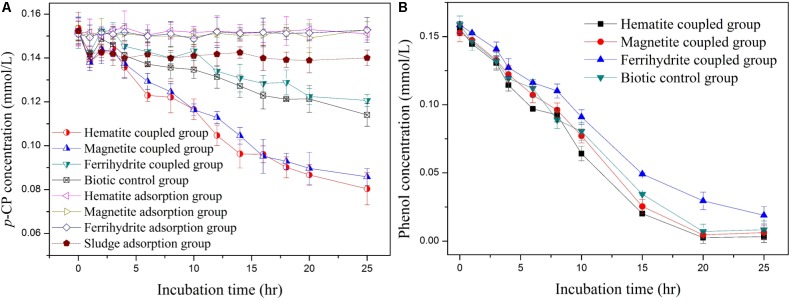
Profiles of *p*-CP dechlorination **(A)** and phenol **(B)** in iron-oxides coupled group, biomass adsorption control group, iron-oxides adsorption control group, and biotic control group.

Three iron-oxide nanoparticles (hematite, magnetite, and ferrihydrite) within similar size range were synthesized successfully, as characterized through XRD (**Supplementary Figure S1**) and SEM (**Supplementary Figure S2**). To ascertain whether the dechlorination of *p*-CP in anaerobic systems could be enhanced by iron-oxide nanoparticles, batch experiments were performed, as shown in **Figure 1A**. In the biotic control system, *p*-CP concentrations decreased from initial 0.152 ± 0.008 mmol/L to 0.114 ± 0.007 mmol/L after incubation time of 25 h. In the biodegradation system coupled with hematite and magnetite, *p*-CP concentrations decreased from initial 0.148 ± 0.007 mmol/L and 0.152 ± 0.006 mmol/L to 0.081 ± 0.008 mmol/L and 0.085 ± 0.008 mmol/L after incubation time of 25 h, which were significantly lower than those in the biotic control system. However, in the biodegradation system coupled with ferrihydrite, the concentrations of residual *p*-CP were always higher than those in the biotic control system at all incubation time. *p*-CP removal was ignorable in iron-oxides adsorption control groups, suggesting that *p*-CP removal through adsorption by hematite, magnetite, and ferrihydrite was insignificant under iron-oxide nanoparticle dosage of 100 mg/L. In addition, less than 10% of *p*-CP could be removed in the biomass adsorption control group within 25 h, indicating insignificant adsorption of *p*-CP by biomass inoculated in the biodegradation system.

As is well-known, *p*-CP could be reductively transformed to phenol through dechlorination under anaerobic condition (Wen et al., 2013). However, phenol detected was negligible during the whole incubation period of 25 h in all incubation system, which could be attributed to the biodegradation of phenol under anaerobic condition. In order to confirm the removal of phenol through biodegradation, phenol removal performance in anaerobic system was investigated. As indicated in **Figure 1B**, 0.156 mmol/L phenol added into anaerobic system could be completely removed within the incubation time of 25 h in all biodegradation system, except for the system coupled with ferrihydrite. Therefore, it could be proposed that in the anaerobic system, *p*-CP could be reductively transformed to phenol, which could be easily removed through anaerobic biodegradation.

### Microbial Metabolism at the Presence of Iron-Oxide Nanoparticles

**FIGURE 2:**
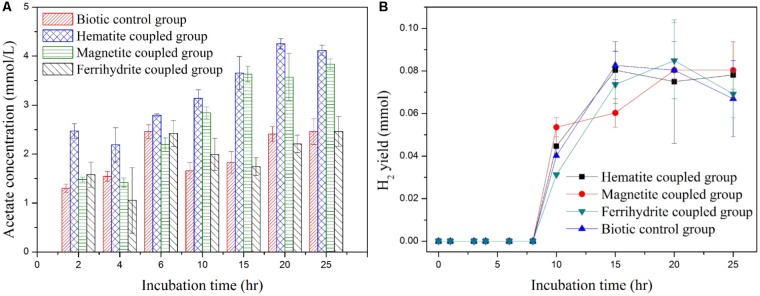
Acetate yield **(A)** and H_2_ yield **(B)** in iron-oxides coupled systems.

Microbial metabolism in the anaerobic system coupled with iron-oxide nanoparticles was compared with that in the control biotic system. It is well-known that in anaerobic system acetate and hydrogen gas are the two main metabolic intermediates, which could act as the electron donor for dechlorination (Scherr et al., 2011; Zhang et al., 2013). As indicated in **Figure 2A**, for the anaerobic system coupled by hematite and magnetite, acetate accumulation after 25 h was as high as 4.12 ± 0.11 and 3.83 ± 0.11 mmol/L, respectively, which was significantly higher than that in the biotic control system. According to Lu et al. (2018), the high acetate accumulation at the presence of magnetite and hematite could be attributed to the promotion role of magnetite and hematite toward the activity and abundance of acidogenic bacteria. However, acetate accumulation in the anaerobic system coupled by ferrihydrite was insignificant at all incubation time. H_2_ production in all anaerobic systems was well below 0.1 mol (**Figure 2B**), probably due to the relatively high ORP and the inhibition effect of *p*-CP in the influent. Considering that acetate was an effective electron donor for the reduction process, the increased production of acetate in the anaerobic system coupled by hematite and magnetite could be beneficial for the dechlorination (Quan et al., 2015).

**FIGURE 3:**
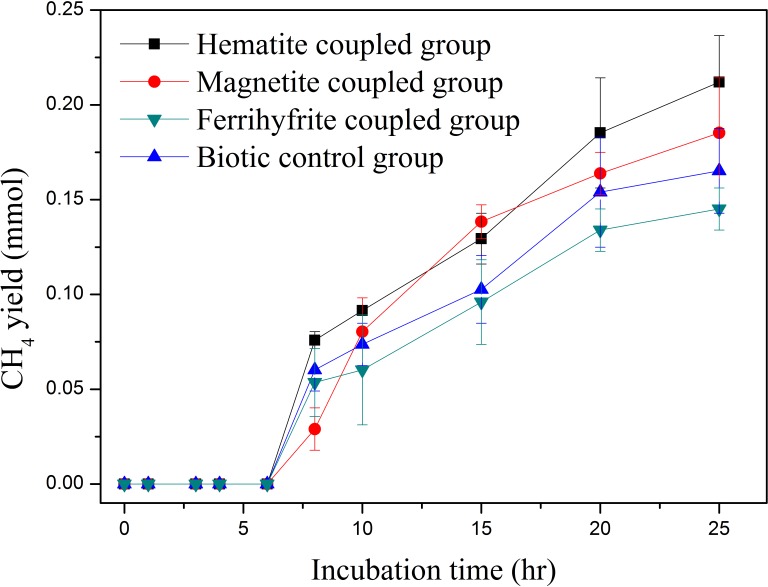
CH_4_ yield in iron-oxides coupled systems.

CH_4_ yields in anaerobic system coupled with hematite and magnetite were 0.21 ± 0.02 and 0.19 ± 0.03 mmol after incubation time of 25 h, as compared to 0.17 ± 0.02 mmol in the biotic control system (**Figure 3**). However, CH_4_ yield in anaerobic system coupled with ferrihydrite was always lower than that in the biotic control system at any incubation time (**Figure 3**). The higher CH_4_ yields in anaerobic system coupled with hematite and magnetite could be attributed to the increased accumulation of acetate at the presence of hematite and magnetite (**Figure 2A**). In our study, the positive effect on methane production by hematite and magnetite were most likely associated with the promotion of direct interspecies electron transfer–mediated syntrophic interactions. Zhuang et al. (2015) proposed that the electrical conductivity of hematite and magnetite was the key factor to accelerate syntrophic metabolism, resulting in facilitated dechlorination, acidogenesis and methanogenesis in anaerobic system.

### ORP and pH Profiles at the Presence of Iron-Oxide Nanoparticles

**FIGURE 4:**
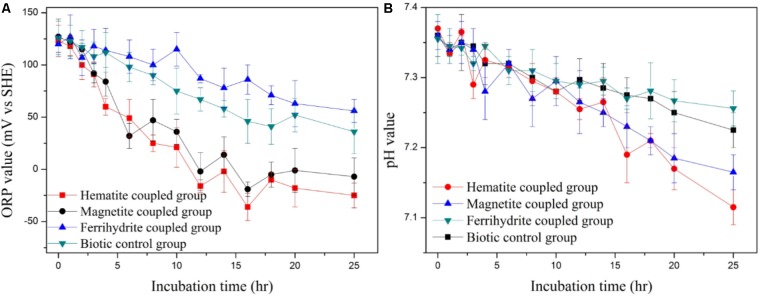
Profiles of ORP value **(A)** and pH value **(B)** in iron-oxides coupled systems.

Oxidation–reduction potential plays a key role in anaerobic metabolism process such as syntrophic acetogenesis, methanogenesis and dechlorination (Zhang Y. et al. (2012)). As shown in **Figure 4A**, the ORP in the hematite and magnetite coupled biosystem decreased from initial 123 ± 15 mV and 127 ± 17 mV to -25 ± 12 mV and -7 ± 18 mV [vs. Standard Hydrogen Electrode (SHE)] at incubation time of 25 h, which was obviously lower than that in the biotic control system. On the contrary, the ORP values in the anaerobic system coupled by ferrihydrite was always higher than those in the biotic control system. Lower ORP value means a better reductive environment, which could exert a positive effect on the reduction of various pollutant such as *p*-CP (Huang et al., 2012; Murali et al., 2013). A previous work showed that both acidogenesis and activity of fermentative bacteria could be effectively improved by lowering ORP (Liu et al., 2012).

As indicated in previous study, pH is one of the most important parameters affecting the community structure and activity of anaerobic microorganisms (Li et al., 2013). As indicated in **Figure 4B**, after incubation for 25 h, the pH values in the biosystem coupled by hematite, magnetite and ferrihydrite, and the biotic control system, shifted from initial 7.36 to 7.11 ± 0.06, 7.16 ± 0.06, 7.25 ± 0.05, and 7.22 ± 0.04, respectively. The lower pH in the biosystem coupled by hematite and magnetite could be linked to the increased acetate production in the anaerobic system, which produced the acidity finally.

### Fe Release in the Anaerobic System

**FIGURE 5:**
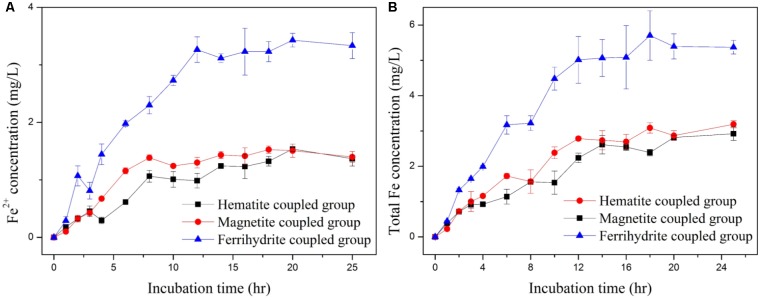
Profiles of Fe^2+^ concentration **(A)** and total Fe concentration **(B)**.

In the anaerobic system coupled by iron-oxide nanoparticles, iron ion could be released from iron-oxide nanoparticles due to the reduction dissimilatory by dissimilating iron-reducing bacteria (Kato et al., 2012). As shown in **Figure 5**, after incubation time of 25 h, Fe^2+^ concentration and total Fe concentration in the anaerobic system coupled with hematite, magnetite and ferrihydrite increased to 1.37 ± 0.13 and 2.92 ± 0.19 mg/L, 1.40 ± 0.09 and 3.19 ± 0.10 mg/L, 3.34 ± 0.23 and 5.37 ± 0.19 mg/L, respectively. The release of both Fe^2+^ and total Fe in the anaerobic system coupled with ferrihydrite was more significant than those in the anaerobic system coupled with hematite and magnetite, indicating that ferrihydrite was susceptible to microbial reduction (Revesz et al., 2016). Meng et al. (2013) indicated that the ferrous ions from iron-oxide corrosion could stimulate the synthesis of key enzymes in the hydrolysis-acidification process, resulting in the accumulation of volatile fatty acids. However, the fact that acetate accumulation was low in the anaerobic system coupled with ferrihydrite ruled out this possibility.

### Synergistic Mechanisms Between Iron Oxides and Anaerobic Microbes

**FIGURE 6:**
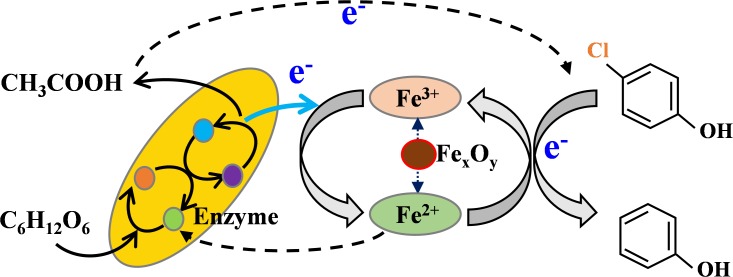
Mechanism involved for enhanced dechlorination in iron-oxide nanoparticles coupled anaerobic system.

Based on the above analysis, the synergy effect between iron oxides and anaerobic sludge for the enhanced dechlorination might be achieved through several ways (**Figure 6**). Firstly, the released Fe^2+^/Fe^3+^ redox couple on the surface of iron oxides could act as the efficient electron mediators, were likely to be capable of accelerating EET (Bae and Lee, 2012). Similar result was also obtained by Wu et al. (2010), where anaerobic transformation of 2,4-dichlorophenoxyacetic acid by an iron-reducing bacterium *Comamonas koreensis* CY01 could be enhanced by Fe (III). It was indicated that *c*-type cytochrome in outer membrane of iron-reducing bacteria could mediate electron transfer from cell to attached iron oxide in cellular metabolism, which resulting in enhanced iron reduction and electron transfer (Zhang W. et al. (2012)). According to these findings, it was reasonable to deduce that the enhanced dechlorination in the coupled system could partially due to the accelerated electron transfer function of Fe^2+^/Fe^3+^ redox couple formed on the surface of iron oxides. Secondly, Fe^2+^ formed through dissimilatory iron reduction could penetrate into the inner part of cells and thus stimulate the synthesis of key enzymes and growth of microbes, particularly methanogens (Zhen et al., 2015). The released Fe^2+^ could decrease electric repulsion and facilitate cell-to-cell interaction between bacteria, which would be further beneficial for the microorganism aggregation in the coupled system (Cruz Viggi et al., 2014). Thirdly, both acidogenesis and methanogenesis was significantly stimulated by hematite and magnetite, but not ferrihydrite. Suppressive effect of ferrihydrite on acidogenesis and methanogenesis has been previously indicated by Kato et al. (2012), who attributed this phenomenon to the high redox potential of ferrihydrite (-100 to +100 mV vs. SHE). Competitive inhibition of dechlorination and methanogenesis by dissimilatory iron reduction for the electron donor such as acetate occurred easily, especially at the presence of ferrihydrite with higher redox potential. What’s more, direct inhibition of methanogenesis by ferric iron was indicated previously by Van Bodegom et al. (2004), which could be another reason for suppressive effect by ferrihydrite. Due to the generation of acetate acid, a more reductive environment could be created by lowering both ORP and pH, especially at the presence of hematite and magnetite, resulting in the significant enhancement of dechlorination and methanogenesis.

### Implication of This Work

Compared to the biotic control system and adsorption control system, *p*-CP removal was significantly improved in the anaerobic system coupled by hematite and magnetite (**Figure 3**). Due to the accumulation of acetate acid, ORP and pH values in anaerobic system coupled with hematite and magnetite were lower than those in the biotic control system, which would be beneficial for both reductive dechlorination and methanogenesis (Huang et al., 2012; Murali et al., 2013). Favorable environment for some specific microbial species, such as methanogens and acetogens, could be created by increasing acetic acid production and lowering the ORP. What’s more important, the presence of hematite and magnetite facilitated electric syntrophy among microorganism, which is also helpful for both reductive dechlorination and methanogenesis (Baek et al., 2016). The efficient reduction of *p*-CP in the anaerobic system coupled by hematite and magnetite would result in a significant improvement of biodegradability and reduction of toxicity (Bae and Lee, 2012). The release of Fe^2+^ and total Fe within 25 h in the anaerobic system coupled by hematite and magnetite was generally below 3 mg/L (**Figure 5**), suggesting the slow dissolution rate of hematite and magnetite. The low consumption of iron oxides leads to easy maintenance and low operating cost. In addition, ferrous iron at low concentration was beneficial for the growth of microorganisms (Pariona et al., 2016).

In previous studies, zero-valent iron (ZVI) was often chosen as the additive of the anaerobic systems in order to enhance echlorination efficiency. For example, the combined ZVI-UASB system treating chloronitrobenzene-containing wastewater showed better shock resistance capacity, higher H_2_/CH_4_ production rate and higher dechlorination efficiency as compared to the control UASB system (Zhu et al., 2015). Chen et al. (2012) confirmed the feasibility of nanoscale ZVI supplement to enhance microbial reductive dechlorination of trichloroethylene (TCE). However, the ZVI particles are unstable and can aggregate easily, which may limit its dispersibility in aqueous solutions and present challenges for *in situ* environmental remediation (Zhang et al., 2018). Iron oxides, such as magnetite and hematite, are much more stable than ZVI, which is beneficial for full-scale application. Besides, the prices of the powdered magnetite and hematite were ddd110 and ddd60 per ton, respectively, which were rather cheap as compared to ZVI (ddd68,000–128,000 per ton). Therefore, the addition of powdered magnetite and hematite into anaerobic systems is not only practically but also economically more viable, as compared with the ZVI. Nowadays, coupling of hematite and magnetite into bench-scale UASB has been developed in our laboratory for the treatment of wastewater containing *p*-CP. The interaction between iron oxides and microorganisms, as well as the dynamic change of microbial population after long-term operation, will be investigated in our future study.

## Conclusion

In this study, anaerobic system coupled with various iron-oxide nanoparticles were investigated to verify the enhanced dechlorination by iron-oxides. Both hematite and magnetite were found to be stimulative, whereas ferrihydrite was found to be suppressive. The anaerobic system coupled with hematite and magnetite showed high dechlorination ability, which could be attributed to significant acetate accumulation, lower ORP and pH values, and enhanced electron transfer by Fe^2+^/Fe^3+^ redox couple on the iron oxide surface. The anaerobic system coupled with iron-oxides such as hematite and magnetite could be a promising alternative to the conventional anaerobic reduction process for the removal of chlorination aromatic compounds from wastewater.

## Author Contributions

XJ, XL, and JS designed the experiments. XJ, YC, and CO carried out the experiments. CH, WH, XS, JL, and LW analyzed the experimental results. XJ, YC, and JS wrote and revised the manuscript. All authors agree to submit the work to Frontiers in Microbiology, and agree to be accountable for all aspects of the work in ensuring that questions related to the accuracy or integrity of any part of the work are appropriately investigated and resolved.

## Conflict of Interest Statement

The authors declare that the research was conducted in the absence of any commercial or financial relationships that could be construed as a potential conflict of interest.
