# Supplementary material for: Promotion of Para-Chlorophenol Reduction and Extracellular Electron Transfer in an Anaerobic System at the Presence of Iron-Oxides
Source: Front Microbiol. 2018 Aug 30;9:2052. doi: 10.3389/fmicb.2018.02052 (PMC6125335; doi:10.3389/fmicb.2018.02052)
Supplement: Supplementary file 1 [file Table_1.DOC]

**Supplementary Information**

**For**

**Promotion of *para*-chlorophenol reduction and extracellular electron transfer in an anaerobic system at the presence of iron-oxides**

Xinbai Jiang^a^, Yuzhe Chen^a^, Chen Hou^a^, Xiaodong Liu^a,*^, Changjin Ou^b^, Weiqing Han^a^, Xiuyun Sun^a^, Jiansheng Li^a^, Lianjun Wang^a^, Jinyou Shen^a,*^

^a^Jiangsu Key Laboratory of Chemical Pollution Control and Resources Reuse, School of Environmental and Biological Engineering, Nanjing University of Science and Technology, Nanjing 210094, Jiangsu Province, China

^b^School of Chemistry and Chemical Engineering, Nantong University, Nantong 226019, Jiangsu Province, China

Corresponding author: ^*^Xiaodong Liu, Tel./Fax: +86 25 84315319, E-mail address: liuxd@mail.njust.edu.cn; ^*^Jinyou Shen, Tel./Fax: +86 25 84303965, E-mail address: shenjinyou@mail.njust.edu.cn

**Characterization of iron-oxide nanoparticles**

The as-synthesized iron-oxide nanoparticles were characterized through XRD, as shown in Fig. S1. The XRD patterns of the obtained three iron-oxide nanoparticles corresponded well with those of iron-oxide nanoparticles reported. The hematite sample exhibited three wide peaks centered at 2θ of 32^o^, 35^o^ and 54^o^ (JCPDS card No. 89-598). All the diffraction peaks of the magnetite sample could be irreproachably indexed to a typical cubic inverse spinel phase of magnetite structure according to JCPDS card No. 89-0688 and No. 76-1849. In addition, no other peaks could be observed, indicating the prepared magnetite was highly purified and well crystallized. The XRD pattern of the ferrihydrite sample was similar with that reported by Pariona et al. (2016), indicating the successful synthesis of ferrihydrite in this study. Both SEM and TEM was performed to characterize morphology and crystal structure of as-synthesized iron-oxide nanoparticles. The as-synthesized hematite showed polyhedral morphology (Fig. S2a and S2b), the single particle has a size in the range of 35-45 nm, which was similar with the hematite nanoparticles prepared by Han et al. (2011). The octahedral morphology of the magnetite highly resembled the sample synthesized by Zhang et al. (2009), with the average size well controlled at about 25-35 nm (Fig. S2c and S2d). According to Fig. S2e and S2f, the ferrihydrite sample displayed quantities of needle-like particles with the length at about 40-60 nm. Therefore, polyhedral hematite, octahedral magnetite and needle-like ferrihydrite within similar size were synthesized successfully in this study.


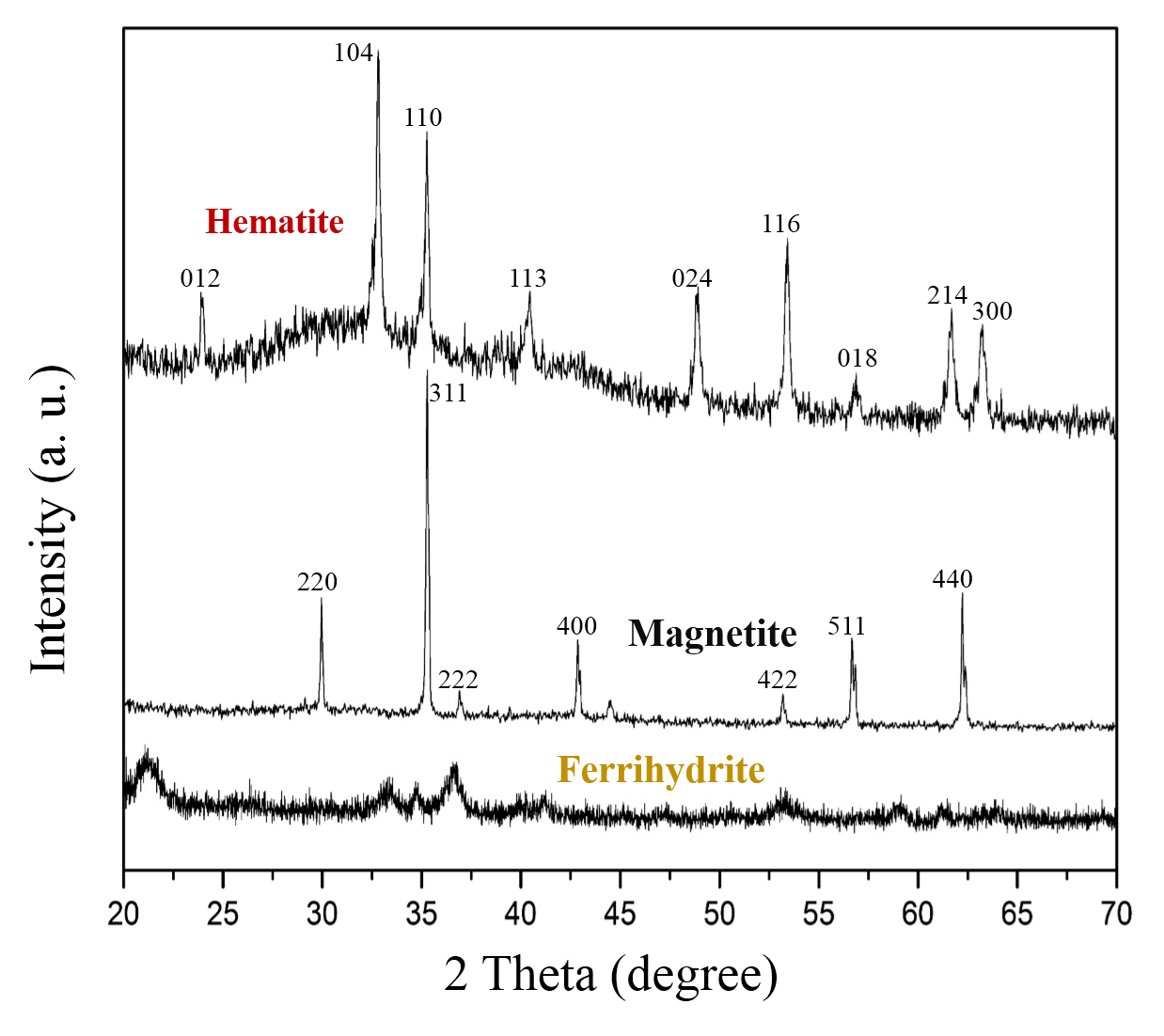


Figure S1 Typical XRD patterns of hematite, magnetite and ferrihydrite nanoparticles.


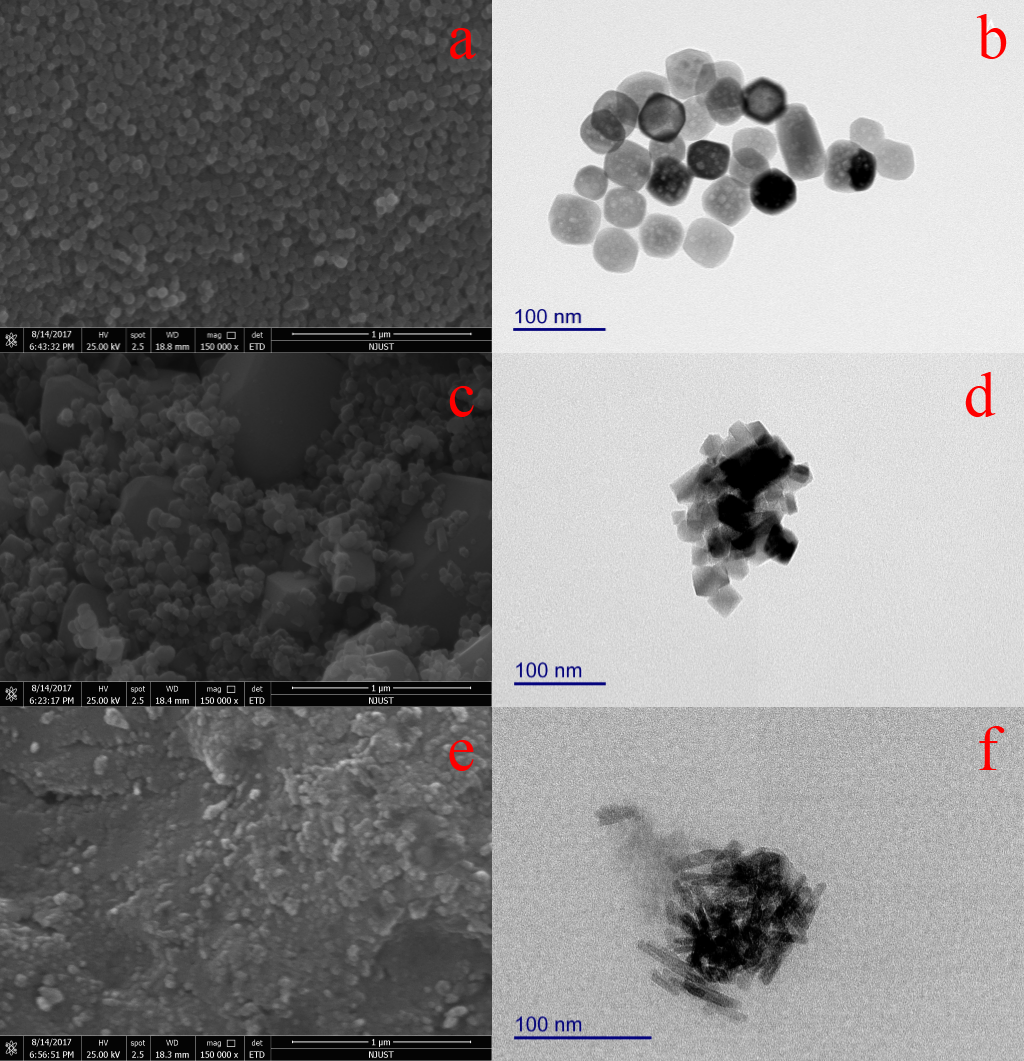


Figure S2 Typical SEM and TEM images of hematite (a, b), magnetite (c, d), ferrihydrite (e, f).

**References**

Pariona, N., Camacho-Aguilar, K.I., Ramos-González, R., Martinez, A.I., Herrera-Trejo, M., Baggio-Saitovitch, E. (2016). Magnetic and structural properties of ferrihydrite/hematite nanocomposites. *J. Magn. Magn. Mater*. 406, 221-227. doi:10.1016/j.jmmm.2016.01.001.

Han, H., Cui, M., Wei, L., Yang, H., Shen, J. (2011). Enhancement effect of hematite nanoparticles on fermentative hydrogen production. *Bioresour. Technol.* 102, 7903-7909. doi:10.1016/j.biortech.2011.05.089.

Zhang, S., Zhao, X., Niu, H., Shi, Y., Cai, Y., Jiang, G. (2009). Superparamagnetic Fe_3_O_4_ nanoparticles as catalysts for the catalytic oxidation of phenolic and aniline compounds. *J. Hazard. Mater.* 167, 560-566. doi:10.1016/j.jhazmat.2009.01.024.
